# Transcriptome Profiling and Physiological Studies Reveal a Major Role for Aromatic Amino Acids in Mercury Stress Tolerance in Rice Seedlings

**DOI:** 10.1371/journal.pone.0095163

**Published:** 2014-05-19

**Authors:** Yun-An Chen, Wen-Chang Chi, Ngoc Nam Trinh, Li-Yao Huang, Ying-Chih Chen, Kai-Teng Cheng, Tsai-Lien Huang, Chung-Yi Lin, Hao-Jen Huang

**Affiliations:** 1 Department of Life Sciences, National Cheng Kung University, Tainan, Taiwan, ROC; 2 Department of Biological Sciences, National Sun Yat-Sen University, Kaohsiung, Taiwan, ROC; National Taiwan University, Taiwan

## Abstract

Mercury (Hg) is a serious environmental pollution threat to the planet. The accumulation of Hg in plants disrupts many cellular-level functions and inhibits growth and development, but the mechanism is not fully understood. To gain more insight into the cellular response to Hg, we performed a large-scale analysis of the rice transcriptome during Hg stress. Genes induced with short-term exposure represented functional categories of cell-wall formation, chemical detoxification, secondary metabolism, signal transduction and abiotic stress response. Moreover, Hg stress upregulated several genes involved in aromatic amino acids (Phe and Trp) and increased the level of free Phe and Trp content. Exogenous application of Phe and Trp to rice roots enhanced tolerance to Hg and effectively reduced Hg-induced production of reactive oxygen species. Hg induced calcium accumulation and activated mitogen-activated protein kinase. Further characterization of the Hg-responsive genes we identified may be helpful for better understanding the mechanisms of Hg in plants.

## Introduction

Mercury (Hg) is considered one of the most harmful metals in the environment. The Maximum Contaminant Level Goals for Hg by the US Environmental Protection Agency have been set at 2 parts per billion [1]. The environmental levels of Hg pollution detected worldwide can be significantly higher. Li et al. [2] reported that sediments from Balkyldak Lake were found to be heavily contaminated, with Hg concentrations in the surface layer reaching up to 1500 mg/kg in Kazakhstan.

At high concentrations, Hg is strongly phytotoxic to plant cells and can induce injury and physiological disorder [3]. Hg accumulates preferentially in roots of several plant species [4]. Therefore, most of the toxic effects are observed in roots. Relatively little is known about the molecular mode of action of Hg stress and the defense responses against it. Hg ions may have toxic reactions with sulfhydryl groups of biomolecules, disrupt cell structure, interfere with cell signaling pathways and displace essential elements. Mercury is detoxified by phytochelatins or their precursor, glutathione, both of which can bind Hg ions to sulfhydryl groups in plants [5]. Furthermore, Hg-induced oxidative damage in plants has been linked to excess production of reactive oxygen species (ROS), which may cause lipid peroxidation, enzyme inactivation and DNA and membrane damage [6]. More recently, suppression subtractive hybridization (SSH) and microarray analysis were used to analyze gene expression profiles of *Arabidopsis* and barley exposed to Hg [7], [8]. Heidenreich et al. [7] and Lopes et al. [8] found that Hg induced some common stress-responsive processes, such as ROS production and enhanced secondary metabolism.

Perception and transmission of stress signals are important aspects of the plant response to environment stress. Modulation in calcium level is sensed by calcium sensors, which trigger a signaling cascade and target the major stress-responsive genes or their transcription factors (TFs) [9]. Protein kinases are crucial in these signaling pathways and have been classified into 7 major phylogenetic groups [10]. By perceiving or sensing extracellular signals, receptor-like kinases (RLKs) activate the downstream signaling pathway by phosphorylating specific targets. RLKs play critical roles in plant development and the response to stress stimuli [11].

Rice (*Oryza sativa* L.) is one of the world’s most widely grown grain crops and also a model plant for molecular biology research. In our previous report, we found that excess Hg increased lipid peroxidation and time-dependently altered total glutathione content and enzymatic activity of antioxidants during Hg stress [6]. Here, we used whole-genome array to analyze the transcriptome response to Hg stress in rice roots. We reveal genes involved in aromatic amino acid, hormone biosynthesis and signaling pathways participating in the Hg stress response.

## Materials and Methods

### Plant Materials

Rice plants (*Oryza sativa* L. cv. TN-67) were grown essentially as described by Yeh et al. [12]. Six-day-old rice seedlings were exposed to 25 µM Hg for 1 to 24 h, and calcium accumulation, mitogen-activated protein kinase (MAPK) activity and microarray analysis of rice roots were as described in the following sections.

### Purification of Total RNA and Semi-quantitative RT-PCR

For microarray analysis and RT-PCR, root samples (100 mg) treated with 25 µM Hg for 1 to 24 h were harvested. Total RNA extraction involved use of the RNeasy Plant Mini kit (QIAGEN, Hilden, Germany) following the manufacturer’s instructions with some modifications. Semi-quantitative RT-PCR and quantitative RT-PCR (qRT-PCR) reactions were performed as previously described [13]. Relative quantification of specific mRNA levels was analyzed using the cycle threshold (Ct) (2^−ΔΔCt^) method [14]. The relative expression levels were normalized using *α-tubulin* (Os03g0726100) as the reference gene. Primer sequences are in Supporting Information (Table S1). Experiments were repeated at least twice and reproducibility was confirmed.

### Microarray Preparation, Data Analysis and Organization

Six-day-old rice seedlings were exposed to 25 µM Hg for short (1 and 3 h) or long (24 h) durations, then RNA was isolated from root tips to examine changes in global gene expression patterns. We pooled RNA isolated after the 2 short-term exposures to maximize gene discovery. RNA from water-treated (control) and Hg-treated roots was used with the Agilent Rice Oligo microarray (4×44 K, custom-made; Agilent Technologies, Palo Alto, CA, USA). RNA labeling and microarray hybridization involved 3 biological replicate samples. Microarray analysis was performed as previously described [15]. For statistical analysis, we excluded genes with signal intensities <100 in all experiments. A significance test with *t* test against zero involved use of GeneSpringGX11 (Agilent Technologies). The Benjamini-Hochberg false discovery rate (FDR) method was used to obtain corrected P values for multiple testing. The fold change for each probe after Hg treatment was calculated by the average of 3 biological replicates. We extracted genes upregulated by Hg by more than two-fold change in expression (FDR cut-off <0.05).

Descriptions of each Hg-responsive gene were annotated by use of the Rice Annotation Project Data Base (http://rapdb.lab.nig.ac.jp) and the TIGR Rice Genome Annotation Resource (http://rice.plantbiology.msu.edu/). In addition, Hg-responsive genes were classified into functional categories by AgriGO functional enrichment analysis [16]. Data on signaling, TF, rice genes encoding protein kinases, TF and transporters were obtained from the Rice Kinase Database (http://rkd.ucdavis.edu), the Database of Rice Transcription Factors (DRTF); http://drtf.cbi.pku.edu.cn/) and the TransportDB (http://www.membranetransport.org), respectively. The microarray data described in this study have been deposited in Gene Expression Omnibus and are accessible by the series accession number [GEO: GSE41719] (http://www.ncbi.nlm.nih.gov/geo/query/acc.cgi?acc=GSE41719). Gene expression changes were depicted by use of MapMan v3.5.1 [17].

### Detection of Calcium and ROS in Rice Roots

Rice roots of 6-day-old seedlings were labeled with 10 µM Oregon Green 488 BAPTA-1 (Molecular Probes, Invitrogen, Carlsbad, CA) or 10 µM 5-(and-6)-chlormethyl-2’,7’-dichlordihydrofluorescein diacetate (CM-H_2_DCF-DA) for 30 min, then with 25 µM Hg for 1∼3 h. Fluorescence images were visualized by confocal microscopy (EZ-C1; Nikon, Tokyo, Japan) with the 488-nm laser line of an Ar laser (2-mW optical fiber output; 500 to 530 nm). Exposure times were equal for all samples.

### Immunoblot Analysis and in-gel Kinase Activity Assay

The proteins from soluble extracts (15 µg) were separated by 10% SDS-PAGE. Immunoblot and in-gel kinase activity analyses were as described [12]. All experiments were repeated at least twice.

### High-Performance Liquid Chromatography (HPLC) of Free Aromatic Amino Acids

Plant extracts were obtained by grinding 200 mg rice roots (shock-frozen in liquid nitrogen) for about 1 min in a mortar after the addition of 100 mg clean sand and 1 ml extraction solvent [50 mM sodium phosphate buffer (pH 6.5)–methanol (97∶3, v/v)]. The mixture was centrifuged for 1 min. The supernatant was filtered through a 0.45-µm filter. An amount of 20 to 50 µl of this clear solution was immediately injected for HPLC separation. The mobile phase used for isocratic elution was prepared by mixing methanol and 50 mM sodium phosphate (monobasic and dibasic) buffer (pH 6.5) in a ratio of 3∶97 (v/v). Before use, the mobile phase was filtered through a 0.45-µm filter and degassed by ultrasonication. The flow rate was 2.0 ml/min; column temperature, 30°C. Compounds were monitored at 215 nm with use of an UV absorbance detector and a Mightysil RP-18 column (150×4.6 mm, 5 µm; Kanto Chemical, Tokyo). Compounds in extracts were identified when the injection of reference compounds (available from Aldrich, Sigma) gave peaks with identical retention times and UV-VIS spectra.

## Results

### Microarray-based Expression Profiling

To begin elucidating the molecular basis of the rice response to Hg, we performed microarray assays with RNA extracted from roots treated with 25 µM Hg after short (pooled from 1- and 3-h treatments) and long (24 h) exposure. In our previous study [6], this 25 µM Hg level reduced root growth to about half the length with control treatment. Transcripts of 1,821 genes were responsive to Hg after short-term exposure and those of 1,482 genes were responsive after long-term exposure (Table S2).

We used AgriGO for GO category enrichment analysis of the 1,263 genes upregulated after short-term exposure and 821 upregulated after long-term exposure (Table 1 and Table S3) [18]. The most significantly enriched GO term was “macromolecule modification”. Other terms included “response to stress”, “transmembrane transport”, “phenylpropanoid metabolic process” and “hormone-mediated signaling pathway”. For molecular function, significant GO terms were “kinase activity” and “transcription factor activity”.

**Table 1:** Gene ontology (GO) analysis of 1,263 genes upregulated with Hg treatment.

GO ID	GO term	Queryitem	Backgrounditem	FDRp-value
	**Biological process**			
**Regulation of biological process**				
GO:0007165	signal transduction	15	106	7.40E-07
GO:0007242	intracellular signaling cascade	13	68	1.50E-07
GO:0009755	hormone-mediated signaling pathway	7	42	5.30E-04
**Cellular component organization**				
GO:0006996	organelle organization	6	28	4.00E-04
**Cellular process**				
*** cellular response to stimulus***				
GO:0070887	cellular response to chemical stimulus	8	43	7.70E-05
*** cellular localization***				
GO:0051649	establishment of localization in cell	5	21	9.30E-04
*** cell communication***				
GO:0007154	cell communication	8	106	3.40E-02
*** transmembrane transport***				
GO:0055085	transmembrane transport	11	12	4.30E-16
*** cellular metabolic process***				
GO:0034645	cellular macromolecule biosynthetic process	78	569	4.30E-31
GO:0009698	phenylpropanoid metabolic process	5	11	2.90E-05
GO:0046417	chorismate metabolic process	6	12	1.60E-06
**Metabolic process**				
*** primary metabolic process***				
GO:0006508	proteolysis	17	126	2.40E-07
*** secondary metabolic process***				
GO:0019748	secondary metabolic process	18	66	5.40E-13
*** macromolecule metabolic process***				
GO:0043412	macromolecule modification	76	265	1.40E-53
GO:0044036	cell wall macromolecule metabolicprocess	11	21	9.30E-12
GO:0016998	cell wall macromolecule catabolicprocess	5	21	9.30E-04
GO:0009059	macromolecule biosynthetic process	78	569	4.30E-31
GO:0009057	macromolecule catabolic process	17	174	2.00E-05
**Establishment of localization**				
*** establishment of protein localization***				
GO:0015031	protein transport	8	22	3.40E-07
*** transport***				
GO:0006811	ion transport	15	66	1.00E-09
GO:0006812	cation transport	14	65	8.80E-09
GO:0030001	metal ion transport	13	36	2.90E-11
**Localization**				
GO:0033036	macromolecule localization	12	22	4.60E-13
GO:0008104	protein localization	8	22	3.40E-07
**Response to stimulus**				
GO:0009725	response to hormone stimulus	9	105	9.00E-03
GO:0032870	cellular response to hormone stimulus	7	42	5.30E-04
GO:0009628	response to abiotic stimulus	7	41	4.60E-04
GO:0009607	response to biotic stimulus	13	39	9.20E-11
GO:0006950	response to stress	54	103	4.00E-55
GO:0006952	defense response	15	59	1.90E-10
GO:0042221	response to chemical stimulus	36	133	3.20E-25
GO:0010033	response to organic substance	12	106	1.20E-04
	**Molecular function**			
**Molecular transducer activity**				
GO:0004871	signal transducer activity	18	32	9.60E-20
**Transporter activity**				
*** substrate-specific transporter activity***				
GO:0022891	substrate-specific transmembrane transporter activity	14	79	1.20E-07
GO:0015075	ion transmembrane transporter activity	9	68	3.40E-04
GO:0008324	cation transmembrane transporter activity	7	62	5.90E-03
*** transmembrane transporter activity***				
GO:0016820	hydrolase activity, acting on acid anhydrides, catalyzing transmembrane movement of substances	7	17	8.60E-07
GO:0042626	ATPase activity, coupled to transmembrane movement of substances	7	17	8.60E-07
GO:0022804	active transmembrane transporter activity	18	56	2.00E-14
GO:0015291	secondary active transmembrane transporter activity	9	24	4.00E-08
GO:0015399	primary active transmembrane transporter activity	8	25	1.00E-06
**Antioxidant activity**				
GO:0004601	peroxidase activity	12	68	1.10E-06
**Transcription regulator activity**				
GO:0003700	transcription factor activity	40	116	1.70E-32
**Catalytic activity**				
*** oxidoreductase activity***				
GO:0004497	monooxygenase activity	23	47	3.30E-23
GO:0051213	dioxygenase activity	5	7	1.50E-06
*** transferase activity***				
GO:0004672	protein kinase activity	59	235	1.30E-38
GO:0004674	protein serine/threonine kinase activity	53	204	1.20E-35
GO:0004713	protein tyrosine kinase activity	8	109	3.80E-02
GO:0016301	kinase activity	67	261	3.10E-44
*** hydrolase activity***				
GO:0004568	chitinase activity	7	21	4.00E-06
	**Cellular component**			
**Macromolecular complex**				
GO:0043234	protein complex	19	133	6.60E-08
GO:0008287	protein serine/threonine phosphatasecomplex	5	14	2.40E-04

FDR, false discovery rate.

**Figure 1:**
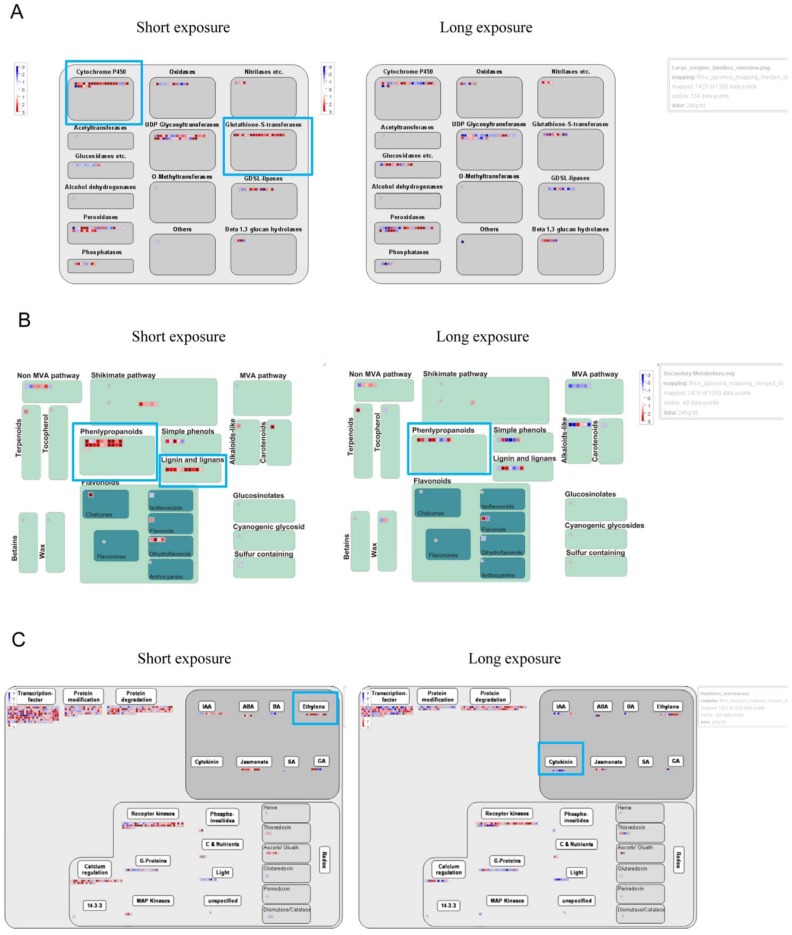
Large enzyme family, secondary metabolism and phytohormone genes were up- or downregulated with Hg stress. MapMan was used to visualize large enzyme family (A), secondary metabolism (B), and phytohormone (C) genes up- or downregulated with short- or long-term 25-µM Hg treatment. Each BIN or subBIN is represented as a block, with each transcript displayed as a square in red for transcripts upregulated or blue for transcripts downregulated.

These findings were supported by more specific comparison of metabolism by use of MapMan [19]. During short-term exposure, the largest family of related enzymes that responded to Hg included cytochrome P450 and glutathione-S-transferase (Figure 1A). Hg stress upregulated secondary metabolism-related genes, which participate substantially in synthesis of phenylpropanoid, lignin and lignans, simple phenols, and flavonoids (Figure 1B). RT-PCR validated the microarray findings (Figure S1 in File S1).

### Polysaccharide and Cell-wall Metabolism Genes Regulated by Hg Stress

Among the 639 cell-wall–response network genes, 19 and 19 were upregulated and 24 and 32 were downregulated with short- and long-term Hg exposure, respectively (Table S4). Cell-wall–related genes upregulated under Hg stress predominantly belonged to expansions (EXPs), yieldins (GH18) and glycoside hydrolases 9 (GH9).

### Expression Profiles of Transporter-associated Genes Regulated by Hg Stress

Among 1,286 transporter-related genes, 78 and 56 were upregulated and 25 and 48 were downregulated with short- and long-term Hg exposure, respectively (Table S5 and S6). Transporter-related genes upregulated with short-term Hg exposure predominantly belonged to the ATP-dependent transporters. Transporter-related genes downregulated with short-term Hg exposure predominantly belonged to the nucleobase:cation symporter-1 (NCS1), sulfate permease (SulP) and oligopeptide transporter (OPT) genes. Transporter-related genes downregulated with long-term Hg exposure predominantly belonged to the proton-dependent oligopeptide transporter (POT) genes.

### Expression Profiles of Phytohormone-related Genes Regulated by Hg Stress

Among 324 phyotohormone-related genes, 19 and 12 were upregulated and 2 and 12 were downregulated with short- and long-term Hg treatment, respectively (Table S5 and S7). Transcripts for 5 ethylene (ET) synthesis genes–OsACS2, OsACO1, OsACO2, OsACO5 and OsACO6– were significantly increased with short-term Hg exposure. Furthermore, transcripts for 5 cytokinin (CK) signaling genes – OsRR1, −3, −4, −6 and −11– were decreased with long-term Hg exposure. MapMan analysis revealed ET synthesis and signaling genes significantly upregulated with short-term Hg exposure (Figure 1C).

### Expression Profiles of Signaling Genes and TFs Regulated by Hg Stress

**Figure 2:**
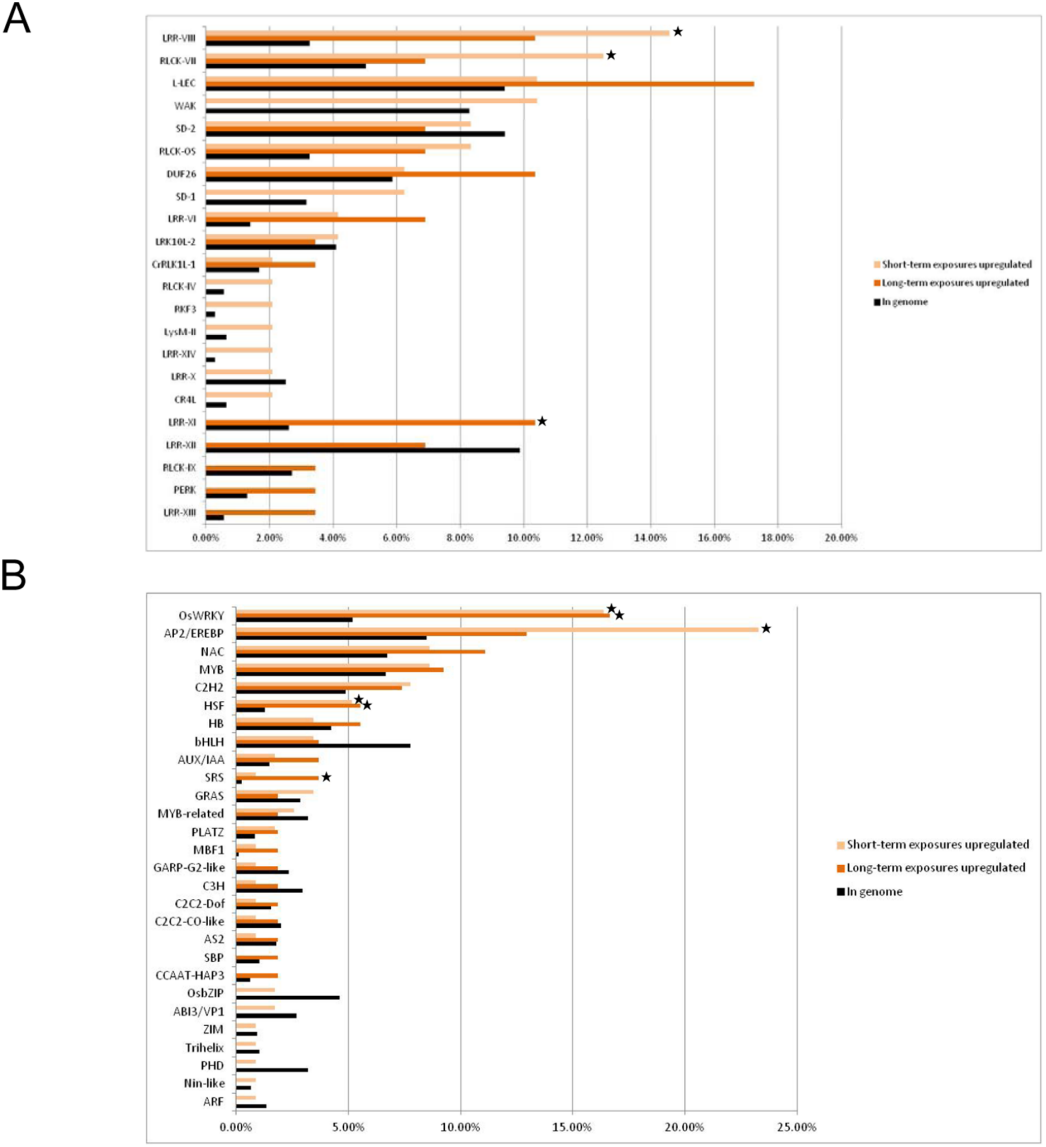
Comparative analysis of genes regulated by short- and long-term Hg exposure and encoding (A) receptor-like kinases (RLKs) and (B) transcription factors (TFs). In each functional category, the genes are grouped according to their regulation by short- or long-term Hg stress. In each protein kinase or TF family, light and dark orange bars represent the proportion of upregulated short- and long-term Hg-regulated genes (fold change ≥2; false discovery rate <0.05), respectively. Black bars indicate the proportion of genes per kinase or TF categories to total number of TFs or kinases in the genome. Fisher’s exact test was used to assess the significance of overrepresented protein kinase and transcription-factor families. Asterisks indicate families significantly overrepresented in the response group (P<0.05).

In total, 87 protein kinase genes were upregulated and 50 were downregulated under Hg stress (Figure 2, Table S8). Nearly all of the Hg-responsive kinases were associated with the RLK family. In total, 48 and 29 protein kinases were upregulated by short- and long-term Hg exposure, respectively (Table S8). LRR-VIII and RLCK-VII subfamilies of the RLK family were significantly upregulated only with short-term Hg exposure (Figure 2, Table S8). The LRR-XI subfamily was significantly upregulated only with long-term Hg exposure.

We found 116 and 54 TFs upregulated with short- and long-term Hg exposure, respectively. Of note, the WRKY and heat shock factor (HSF) families were significantly upregulated with both short- and long-term Hg treatment, and APETALA2/ethylene response factor (AP2/ERF) and SHI-related sequence (SRS) families were regulated only with short- and long-term Hg treatment, respectively. Of note, the HB family was significantly downregulated with both short- and long-term Hg treatment (Figure 2, Table S9).

### Hg Induced Calcium Accumulation and MAPK Activity in Rice Roots

**Figure 3:**
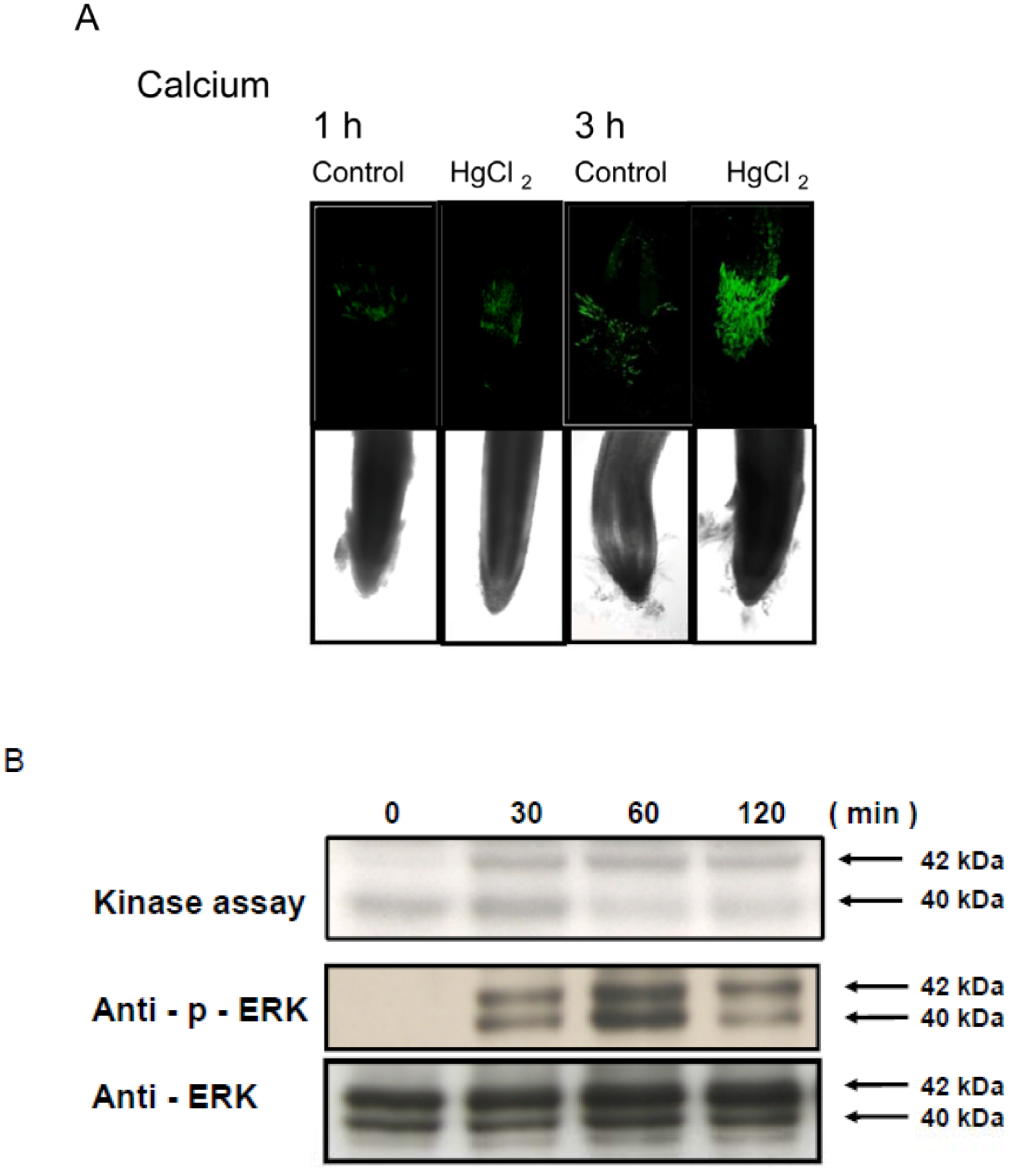
Calcium accumulation and response of mitogen-activated protein kinase (MAPK) activity to Hg by dose and time in rice roots. (A) Root samples were labeled with 10 µM Oregon Green 488 BAPTA-1, a calcium indicator, for 30 min, then treated with 25 µM Hg for various times (1∼24 h). Green fluorescence indicates the presence of calcium. Five control and 5 treated roots showed similar results. Magnification for all images was ×100. Rice roots were treated with (B) 25 µM Hg for various times. A 15-µg aliquot of crude protein was separated by SDS-PAGE and analysed by in-gel kinase activity assay and western blot with anti-phospho-extracellular signal-regulated kinase (ERK) or anti-ERK antibodies. Arrows indicate kinase-active bands.

To determine whether Hg treatment induced calcium accumulation, we treated rice roots with a calcium indicator, Oregon green 488 BAPTA-1, before Hg treatment. Calcium level was significantly increased in root tip regions with 25 µM Hg treatment for 1 or 3 h (Figure 3A). To determine whether MAPK activity was also induced by Hg in rice roots, we performed immunoblot analysis and in-gel kinase activity assay. Two MAPKs of approximately 40 and 42 kD were dose-dependently activated in rice roots challenged with different concentrations of Hg for 1 h (Figure S2 in File S1), with maximal activity at 50 µM. As well, 25 µM Hg rapidly and transiently induced the activation of the 40- and 42-kD MAPK within 30 min, which peaked at 60 min, then began to decrease at 120 min (Figure 3B).

### Hg Induced Free Aromatic Amino Acid Accumulation in Rice Roots

**Figure 4:**
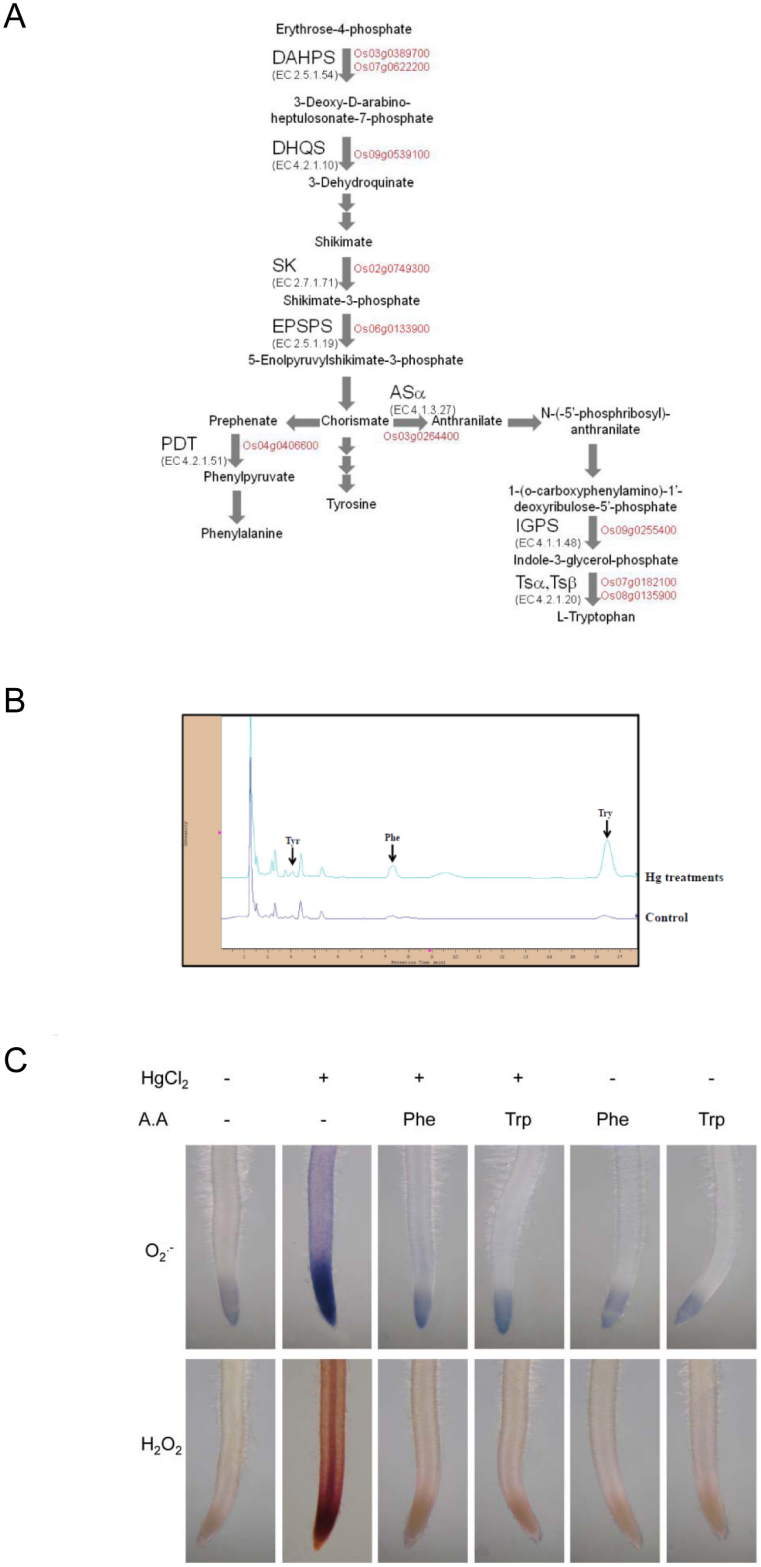
Induction of aromatic metabolites and effect of amino acid treatment to Hg-induced reactive oxygen species (ROS) production in rice roots. (A) Hg-responsive genes involved in the chorismate metabolic process and free aromatic amino acid accumulation in rice roots. Genes in red are upregulated in Hg-treated rice roots. Enzyme abbreviations: ASα anthranilate synthase α subunit; DAHPS 3-deoxy-D-arabino-heptulosonat-7-phosphate synthase; DHQS 3-dehydroquinate synthase; SK shikimate kinase; EPSPS 5-enolpyruvylshikimate 3-phosphate synthase; IGPS, indole-3-glycerol phosphate synthase; PDT, prephenate dehydratase; TSα, tryptophan synthase αsubunit; TSβ, tryptophan synthase βsubunit. (B) HPLC chromatograms of aromatic amino acids Phe, Tyr and Trp in rice root extracts with or without Hg treatment. Column, 250×4.6 mm I.D.; flow-rate, 2.0 ml/min; mobile phase, methanol–50 mM sodium phosphate buffer (pH 6.5) (3∶97, v/v). (C) Effect of aromatic amino acid (Phe, Trp) treatment on 25 µM Hg-induced ROS accumulation in rice roots. Root samples pretreated or not with 100 µΜ aromatic amino acid (Phe, Trp) for 30 min were treated with 25 µM Hg for 0 to 3 h. Superoxide anion and H_2_O_2_ levels were detected by treating roots with nitroblue tetrazolium and 3,3′-diamninobenzidine, respectively.

AgriGO and MapMan analysis revealed a strong effect of Hg on the chorismate metabolic process and aromatic amino acid genes (Phe, Trp, and Tyr) (Figure 4A, Figure S3 in File S1, Table 1). To provide further evaluation of our microarray data, qRT-PCR analysis was carried out to quantify the changes in mRNA expression level. We selected 10 candidate aromatic amino acid genes obtained by microarray analysis (Figure S4 in File S1). We found induction of several aromatic substances (Phe, Trp, and Tyr) at 3 h after Hg treatment (Figure 4B). Among the identified induced substances, Trp level was increased more than five-fold. Also, Phe level was increased about two-fold.

### Effect of Amino Acid Treatment on Hg-Induced ROS Production in Rice Roots

To elucidate the possible relation between the accumulation of free aromatic acids (Phe and Trp) and resistance to Hg, we next tested the role of these aromatic acids in Hg-induced ROS production. Superoxide anion was distributed in root tips of Hg-treated roots; H_2_O_2_ was produced in both root tips and vascular tissues (Figure 4C). Rice seedling roots were pre-treated with or without aromatic acids (Phe and Trp), and ROS generation was assayed in rice roots immediately after exposure to Hg stress. Histochemical staining for superoxide anion and H_2_O_2_ revealed markedly decreased ROS accumulation in roots treated with aromatic acids (Phe and Trp) during Hg stress. Rice seedling roots were pre-treated with or without Trp inhibitor [5-methyltryptophan (5MT)] and ROS level was significantly increased in roots treated with 5MT during Hg stress (Figure S5 in File S1).

## Discussion

Hg toxicity can inhibit plant growth by damaging or disrupting the function of roots [6]. Here, we observed that Hg was toxic to rice roots: 25 µM Hg decreased rice root length. Root-stress–induced morphogenic responses involve changes in phytohormones [20]. In this study, we found that 5 ET synthesis genes were strongly induced with short-term Hg exposure. In addition, the expression of a least 5 genes implicated in CK signaling was downregulated during long-term exposure to Hg stress. Massot et al. [21] reported that increased CK levels preceded Al-induced inhibition of root growth in bean seedlings. Our data indicated downregulated CK signalling with Hg treatment. In general, cell walls become lignified with decreased cell expansion or when the cell is under stress [22]. Our microarray assay revealed induced expression of many lignin genes in rice roots after Hg treatment (Figure 1B). Lignification may be an important step in root growth reduction in stressed plants [23]. Thus, ET, CK and lignification may be involved in Hg-induced inhibition of rice root growth.

Increased ROS levels are an important component of stresses induced by heavy metals [12]. Our previous study indicated that Hg was an oxidative stress agent and may cause increased ROS accumulation, lipid peroxidation and lipoxygenase activity, which may affect cell integrity in rice roots [6]. ROS production may contribute to Hg-induced root growth inhibition. A primary response to the presence of heavy metals in whole living organisms involves the induction of detoxifying enzymes [24]. Soranzo et al. [25] reported that glutathione-S-transferases (GSTs) are a large family of key defense enzymes against heavy-metal toxicity and oxidative stress [26]. In our GO term enrichment analysis, a significant GO term was “glutathione transferase activity”. Metallothioneins (MTs) are known to be involved in ROS scavenging and metal homeostasis [27]. Among the 11 MT genes, 1 and 4 were significantly upregulated with short- and long-term Hg treatment, respectively (Table S10). Ruiz et al. [28] reported that the expression of mouse MT in transplastomic plants increased Hg resistance. Upregulation of GST and MT genes may be important in improving defense against heavy metal stress by upregulating ROS scavengers.

In our GO term enrichment analysis, a significant GO term was “chorismate metabolic process”. In plants, the 3 aromatic amino acids (Phe, Trp, and Tyr) are synthesized from the common precursor metabolite chorismate [29]. Here, we found that Hg significantly upregulated key enzymes for Trp synthesis–anthranilate synthase alpha 2 subunit (Os03g0264400), indole-3-glycerol phosphate synthase (Os09g0255400) and Trp synthase (Os07g0182100 and Os08g0135900) – as well as Phe synthesis – prephenate dehydratase (Os04g0406600). Upon exposure to metals, plants often synthesize a set of diverse metabolites that accumulate to high concentrations; particular amino acids are Phe, Trp, and Tyr [30]. Jo et al. [31] found that the role of Trp in the response to Cu-induced toxicity in yeast may be via the antioxidant properties of Try metabolites or its radical-scavenging activity. Sanjaya et al. [32] found that Trp could confer Cd tolerance. Here, we found that exogenous application of Phe and Trp counteracted Hg-induced toxicity and ROS accumulation in rice roots. We and Lopes et al. [8] found the upregulation of the transcripts related to the abscisic acid (ABA) biosynthesis and signaling pathway under Hg stress. Kang et al. reported that ABA induced Trp synthesis [33]. Phe is the precursor of the phenylpropanoid pathway, which is important for the biosynthesis of lignin and flavonoids [29]. The increase in Phe, Trp, ABA and lignin/flavonoid may play important roles in protecting rice seedlings against Hg toxicity.

In our GO enrichment analysis, a significant term was “transmembrane transport”. Among 78 and 56 transporter-related genes upregulated with short- and long-term Hg treatment, ABC-family and H^+^-PPase-family transporters were significantly regulated, respectively. The ABC group of transporters may facilitate the movement of glutathionylated toxins and other substrates across biological membranes [34]. We found 20 and 10 ABC transporter genes upregulated with short- and long-term Hg exposure, respectively. Thus, ABC transporters, which work in conjunction with other detoxifying systems, are associated primarily with early stages of Hg stress. Among the Hg-responsive genes, 54 secondary transporters were downregulated. The most predominant families were the OPT, POT, and sulfate permease (SulP) families. The OPT and POT families are transporters of diverse peptides [35]. Song et al. [36] found that antisense plants of AtPTR2, an *Arabidopsis thaliana* POT, showed delayed flowering and arrested seed maturation. Therefore, Hg may have a significant effect on inhibiting plant growth by regulating nitrate and peptide transport. In addition, we found that Hg repressed the expression of 3 aquaporin genes (OsPIP2;3, Os04g0521100; OsPIP2;5, Os07g0448400; OsNIP2;1, Os02g0745100) in rice roots (Table S11). Zhang and Tyerman [37] reported that Hg^2+^ ion affected aquaporin activity and blocked the expression of most plant aquaporins [8], [38], [39]. Thus, downregulation of aquaporin genes may be involved in affecting water relations and contribute to Hg toxicity in rice roots.

Understanding the molecular mechanisms of how plant cells monitor and respond to metals is important. Protein kinases are crucial in these signalling pathways. By perceiving or sensing the extracellular signals, RLK activates the downstream signaling pathway by phosphorylating specific targets [40]. Among 48 RLK genes upregulated with short-term Hg treatment, LRR-VIII and RLCK-VII were significantly regulated. The involvement of the LRR-VIII and RLCK-VII subfamilies in stress responses was previously reported [41], [42]. The LRR-VIII subfamily is broadly overrepresented in genes upregulated by most biotic stresses [42]. In addition, we found 3 members of the LRR-XI subfamily significantly upregulated with long-term Hg exposure. However, The LRR-XI subfamily does not seem to respond substantially to most abiotic and biotic stresses [42]. The LRR-XI subfamily members function in development [43]. Recently, Pitorre et al. [44] found that the LRR-XI subfamily is involved in tolerance to oxidative stress in *Arabidopsis* biology. Thus, differential expression of a number of transmembrane receptor kinases with exposure to Hg suggests that multiple receptors belonging to different families may have unique regulatory mechanisms.

We and others demonstrated that environmental stresses activate MAPK signalling pathways and calcium in plants [12], [45]. Previous study showed increased mRNA expression of rice MAPKKK, MAPKK and MAPK genes under various stresses [46]–[48]. Here, we found 5 MAPKKK, 1 MAPKK and 2 MAPK genes with significantly increased mRNA expression under Hg stress. In addition, we demonstrated activation of 40- and 42-kD MAPKs with Hg treatment. Furthermore, Yeh et al. [12] found that calcium is required for MAPK activation. In our study, Hg increased calcium levels in rice roots. These results emphasize a potentially important role for calcium and MAPK signalling in Hg stress responses.

TFs are the important messengers between a perceived stimuli and the induced response. We found the major TFs for HSF and WRKY families significantly upregulated among the TFs responding to short- and long-term exposure in rice. In addition, AP2/ERF and SRS families were regulated only with short- and long-term Hg treatment, respectively. Many studies reported that HSF, WRKY and AP2/ERF play important roles in plant resistance to biotic and abiotic stresses [49]–[51]. Recently, Ishihama et al. [52] reported that MAPK phosphoryates group I WRKY genes. WRKY proteins are involved in various stress signalling pathways [53]. In our array data, 5 group I WRKY TFs – OsWRKY24, −30, −38, −53 and −70– were upregulated under Hg stress. Group I OsWRKY genes may have some role in the Hg-stress response by activating rice MAPKs. Fridborg et al. [54] reported that the SRS family of proteins, including rice SRS1 (Os01g0954500), negatively regulates giberellic acid–induced cell elongation. We found the rice SRS1 (Os01g0954500) and SRS3 (Os06g0712600) genes strongly upregulated with Hg treatment in rice. The SRS family of proteins may be involved in Hg inhibition of rice root growth through a giberellic acid pathway.

In our previous study, we found that Hg induced ROS production. Many studies have demonstrated that the toxic effect of heavy metals (eg, Cu and Cd) may be related to ROS production. Thus, we compared the set of Hg-regulated genes to those regulated by exposure to the heavy metals Cu and Cd and the ROS-generating allelochemical juglone [15], [55]. As compared with Cu, Cd and juglone stress, Hg specifically upregulated one R2R3 Myb TF (MYB-81, Os06g0221000) (Table S9 and S12). Several studies have demonstrated that the Myb TFs play important roles in the regulation of gene expression cope with highly variable environmental stresses [56]. This finding suggests that this R2R3 Myb TF regulates downstream genes playing essential physiological roles under Hg but not general environmental stress.

In conclusion, our data imply that Hg may have a significant effect on the inhibition of root elongation through ET and CKs. In addition, Hg influences amino acid metabolism, peptides and water transport, which is required for the fundamental biological processes of all organisms (Figure S6 in File S1). Detoxification enzymes such as GST and MT protect against Hg toxicity. Physiologically relevant concentrations of free aromatic amino acids (Phe and Trp) might contribute to the plant response to Hg stress by reducing Hg-induced ROS production. Thus, Phe and Trp could be involved in Hg defence. Further studies with Hg-responsive genes and pathway-specific mutants should lead to a more profound understanding of the network of pathways that mediate the plant response to Hg stress.

## Supporting Information

File S1: Figures S1-S6 Figure S1 Verification of microarray data by RT-PCR. Figure S2 HG induced MAPK activity in rice roots treated with 0-50 ?MHG for 1 h. Arrows indicate kinase-active bands. Figure S3 Genes in aromatic amino acids synthesis up- or downregulated with Hg treatment in rice roots. MapMan was used to visualize genes up- or downregulated in aromatic amino acidsynthesis. Each BIN or subBIN is represented as a block, with eachtranscript displayed as a square, colored red for upregulation or blue for downregulation. Figure S4 Time course of Hg effect on expression of genes involved in Trp systhesis. Relative mRNA expression wascalculated via the Livak method (2^-DDCt^). Data are presented as mean relative expression 6 SD for 3 replicate real-time reactions from 3 independent samples. Means with asterisks are significantlydifferent at P, 0.05 level. Figure S5 Effect of Trp inhibitor [5-methyltryptophan (5 MT)] treatment on 25 μM Hg-induced ROS accumulation in rice roots. Root samples pretreated or not with 100 μM 5 MT for 30 min were treated with 25 μM Hg for 0 to 3 h. Green fluorescence indicates the presence of ROS in rice roots. Figure S6 The molecular mode of action of Hg in various cellular processes and response/regulatory pathways in rice. Anoxidative burst at the place of injury generates reactive oxygen species (ROS), which can lead to induced cell death and root growth inhibition. Gene families repressed and activated by Hgare in blue (fold change #0.5) and red (fold change $2), respectively.
(PPTX)

Table S1 Oligonucleotide primers for semi-quantitative RT-PCR and quantitative RT-PCR.
(XLSX)

Table S2 List of regulated genes responding to 25 µM Hg.
(XLSX)

Table S3 Gene ontology analysis of 1,263 genes upregulated with short-term (1- and 3-hr exposure pooled) exposure and 821 genes upregulated with long-term (24-h) exposure to 25 µM Hg.
(XLSX)

Table S4 Expression profiles of cell wall-related genes induced by 25 µM Hg.
(XLSX)

Table S5 Hg-responsive transcripts related to transporter genes and phytohormone-related genes.
(DOCX)

Table S6 Expression profiles of genes associated with transporters with Hg stress.
(XLSX)

Table S7 Expression profiles of phytohormone-related genes induced by Hg stress.
(XLSX)

Table S8 Expression profiles of protein kinase genes induced by Hg stress.
(XLSX)

Table S9 Expression profiles of transcription factors induced by Hg stress.
(XLSX)

Table S10 Expression profiles of metallothioneins induced by Hg stress.
(XLSX)

Table S11 Expression profiles of aquaporin induced by Hg stress.
(XLSX)

Table S12 Summary of Hg-specific upregulated genes.
(XLSX)
