# Supplementary material for: Transcriptome Profiling and Physiological Studies Reveal a Major Role for Aromatic Amino Acids in Mercury Stress Tolerance in Rice Seedlings
Source: PLoS One. 2014 May 19;9(5):e95163. doi: 10.1371/journal.pone.0095163 (PMC4026224; doi:10.1371/journal.pone.0095163)
Supplement: File S1: Figures S1-S6 — Figure S1 Verification of microarray data by RT-PCR. Figure S2 HG induced MAPK activity in rice roots treated with 0-50 ?MHG for 1 h. Arrows indicate kinase-active bands. Figure S3 Genes in aromatic amino acids synthesis up- or downregulated with Hg treatment in rice roots. MapMan was used to visualize genes up- or downregulated in aromatic amino acidsynthesis. Each BIN or subBIN is represented as a block, with eachtranscript displayed as a square, colored red for upregulation or blue for downregulation. Figure S4 Time course of Hg effect on expression of genes involved in Trp systhesis. Relative mRNA expression wascalculated via the Livak method (2-DDCt). Data are presented as mean relative expression 6 SD for 3 replicate real-time reactions from 3 independent samples. Means with asterisks are significantlydifferent at P, 0.05 level. Figure S5 Effect of Trp inhibitor [5-methyltryptophan (5 MT)] treatment on 25 μM Hg-induced ROS accumulation in rice roots. Root samples pretreated or not with 100 μM 5 MT for 30 min were treated with 25 μM Hg for 0 to 3 h. Green fluorescence indicates the presence of ROS in rice roots. Figure S6 The molecular mode of action of Hg in various cellular processes and response/regulatory pathways in rice. Anoxidative burst at the place of injury generates reactive oxygen species (ROS), which can lead to induced cell death and root growth inhibition. Gene families repressed and activated by Hgare in blue (fold change #0.5) and red (fold change $2), respectively. (PPTX) [file pone.0095163.s001.pptx]

## Slide 1
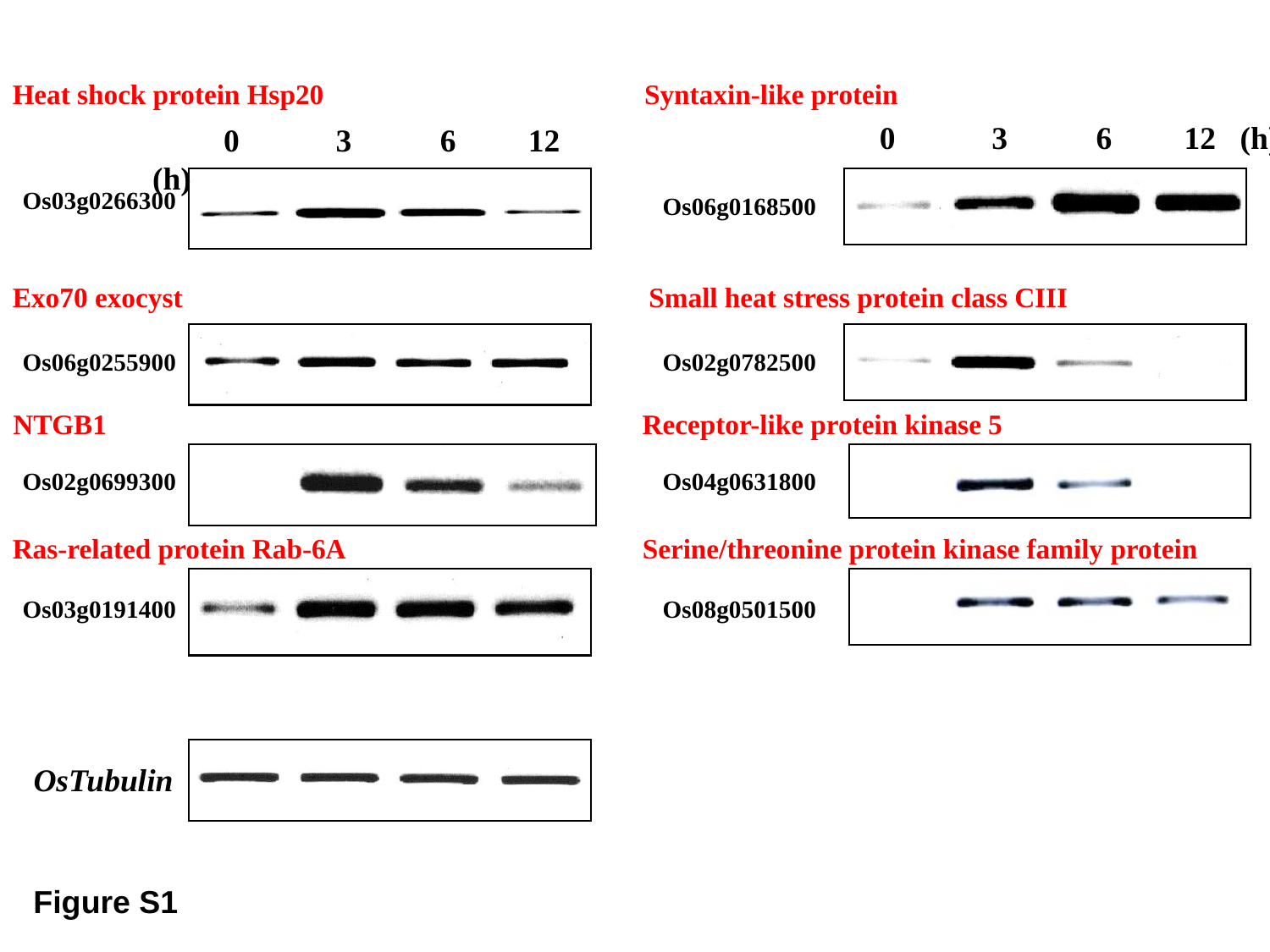

Heat shock protein Hsp20
Syntaxin-like protein
 0 3 6 12 (h)
 0 3 6 12 (h)
Os03g0266300
Os06g0168500
Exo70 exocyst
Small heat stress protein class CIII
Os06g0255900
Os02g0782500
Receptor-like protein kinase 5
NTGB1
Os02g0699300
Os04g0631800
Ras-related protein Rab-6A
Serine/threonine protein kinase family protein
Os08g0501500
Os03g0191400
OsTubulin
Figure S1

## Slide 2
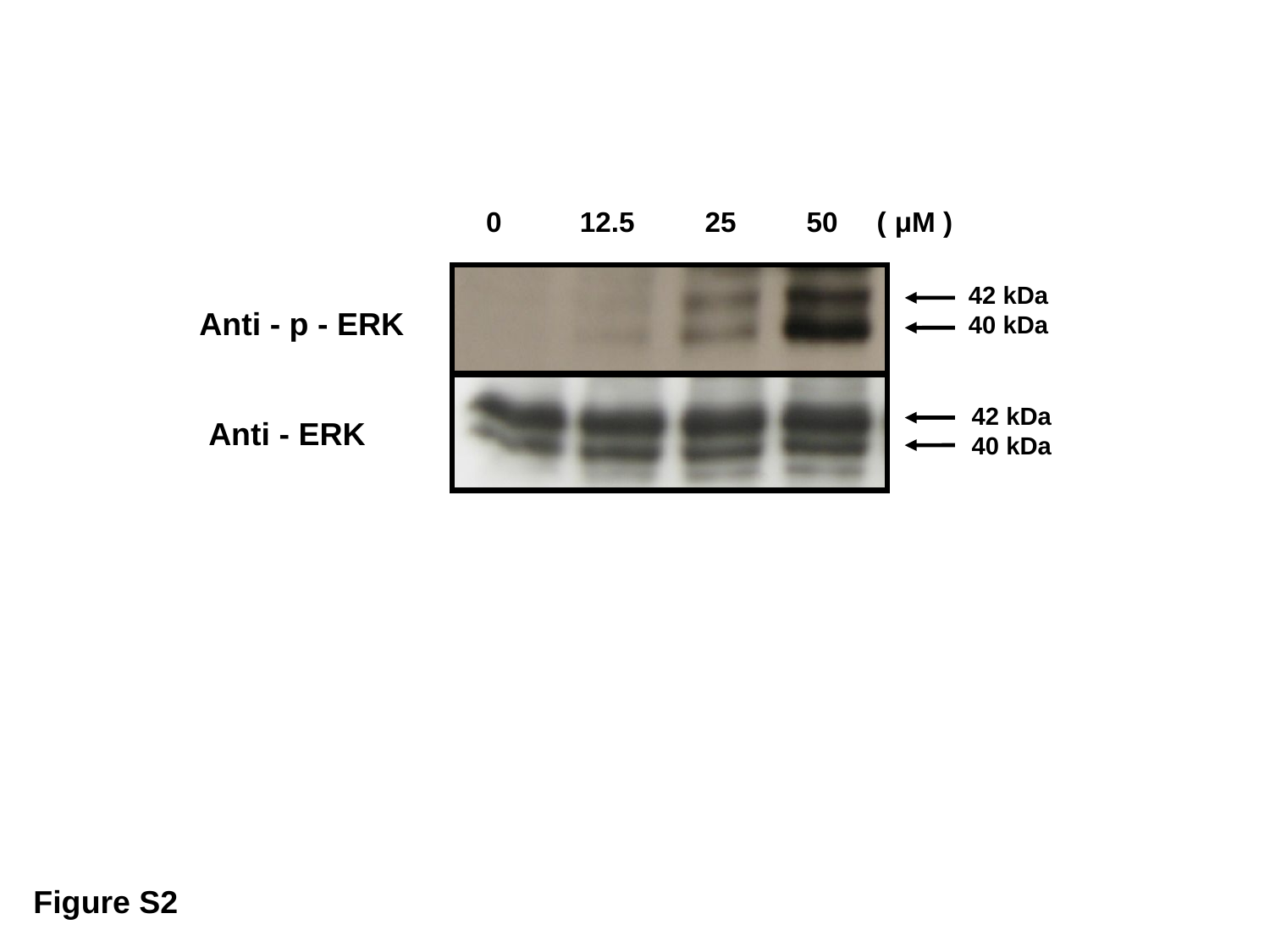

0 12.5 25 50 ( μM )
42 kDa
40 kDa
Anti - p - ERK
42 kDa
40 kDa
Anti - ERK
Figure S2

## Slide 3
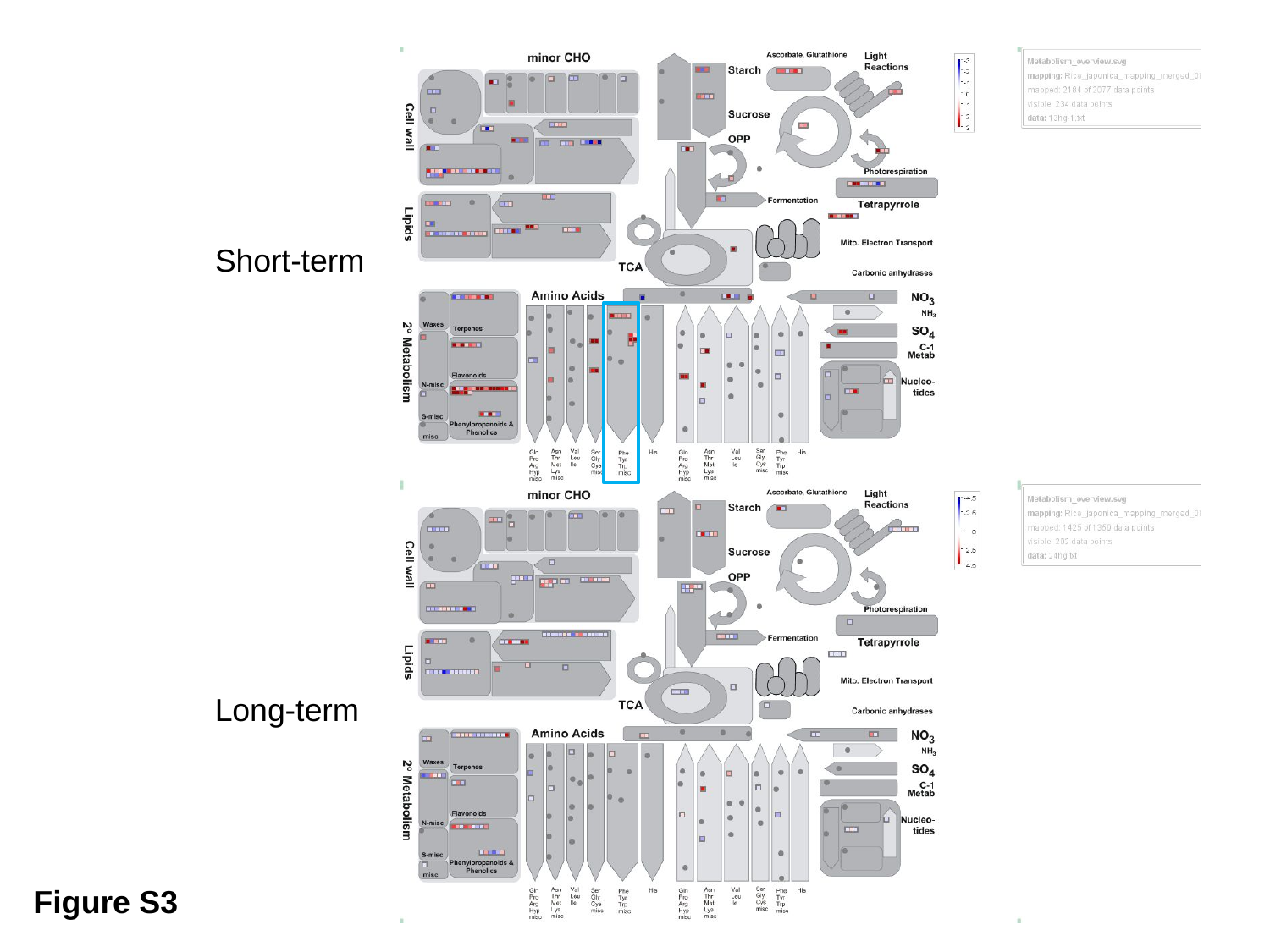

Short-term
Long-term
Figure S3

## Slide 4
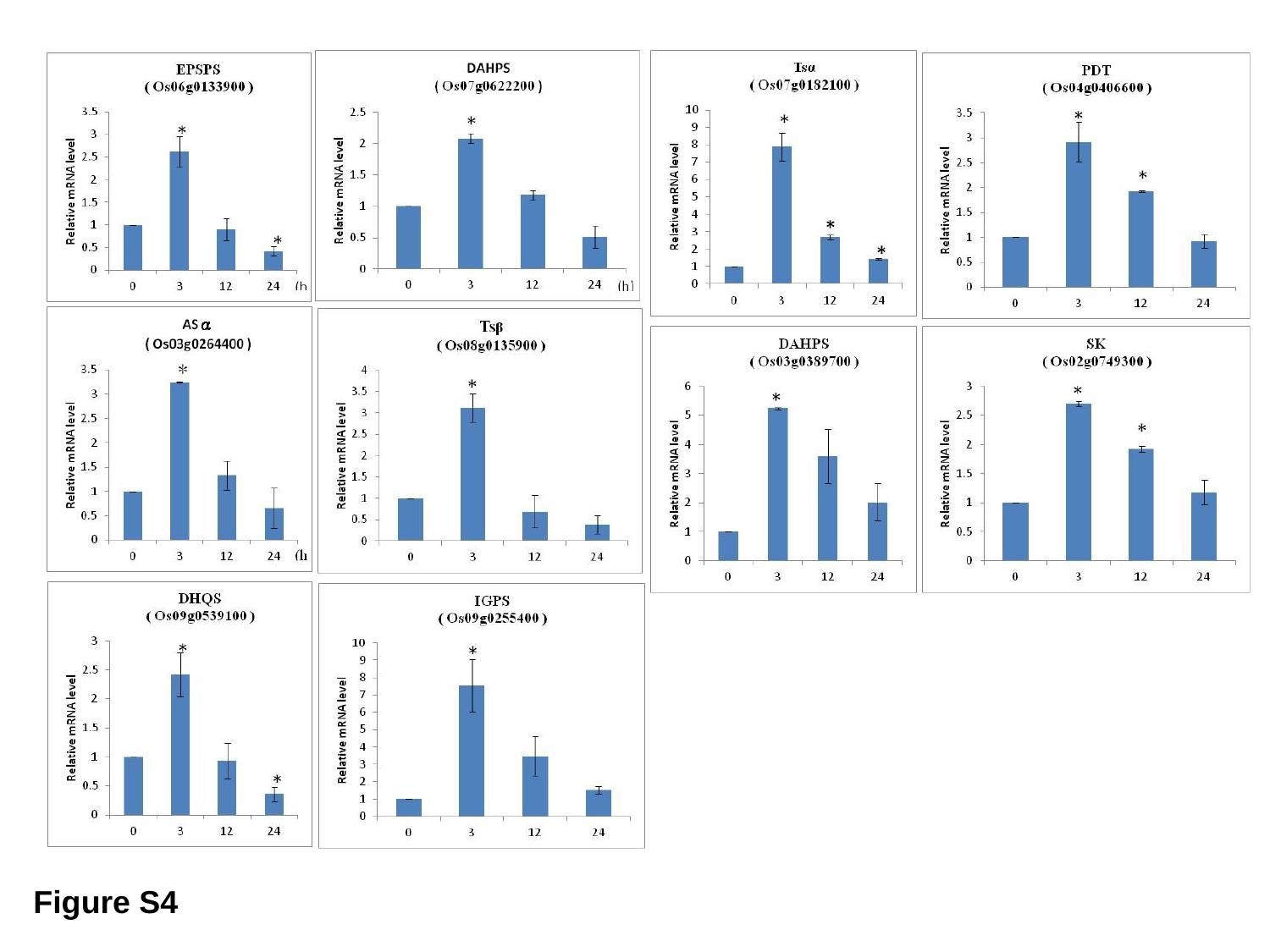

Figure S4

## Slide 5
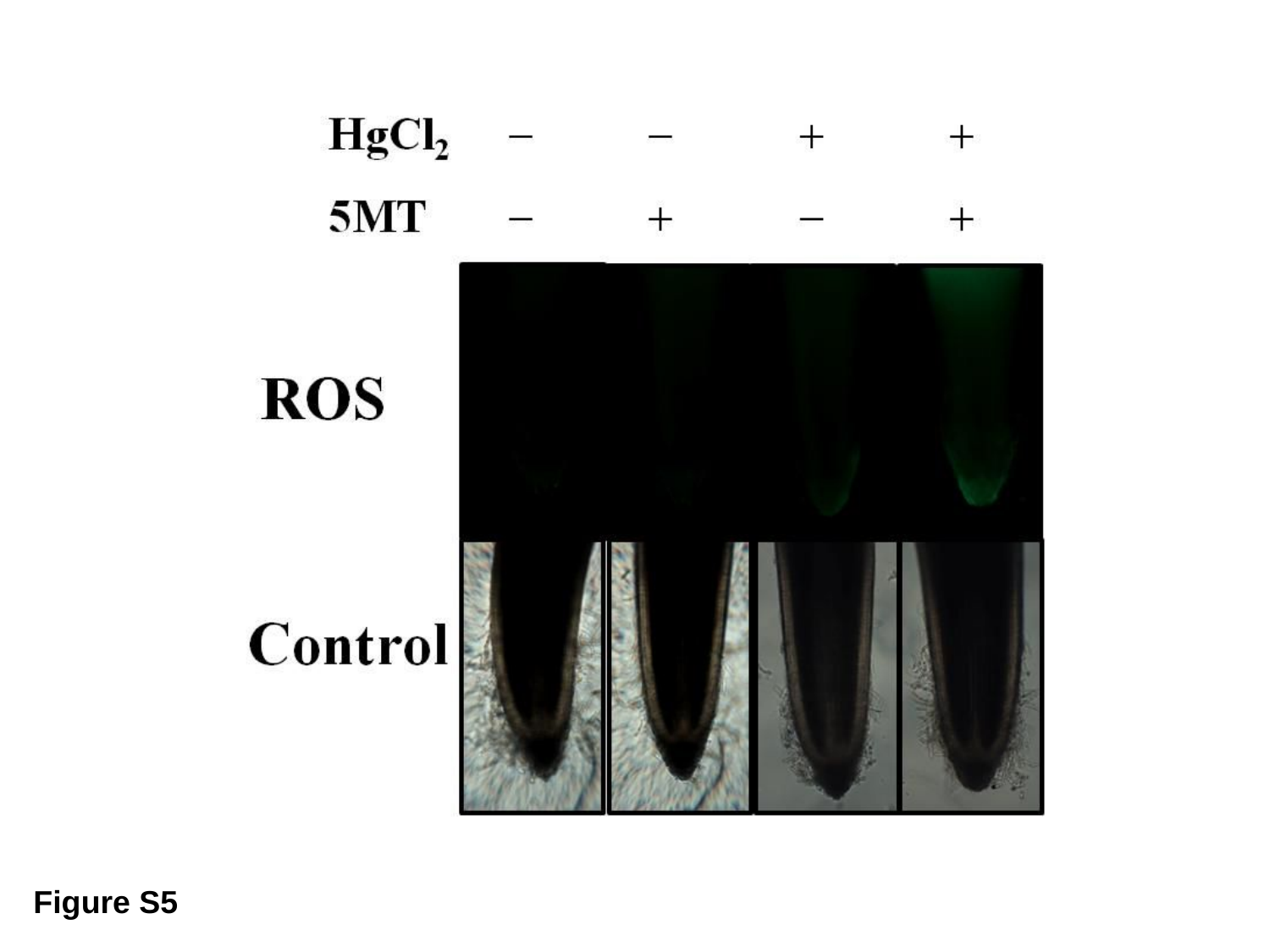

Figure S5

## Slide 6
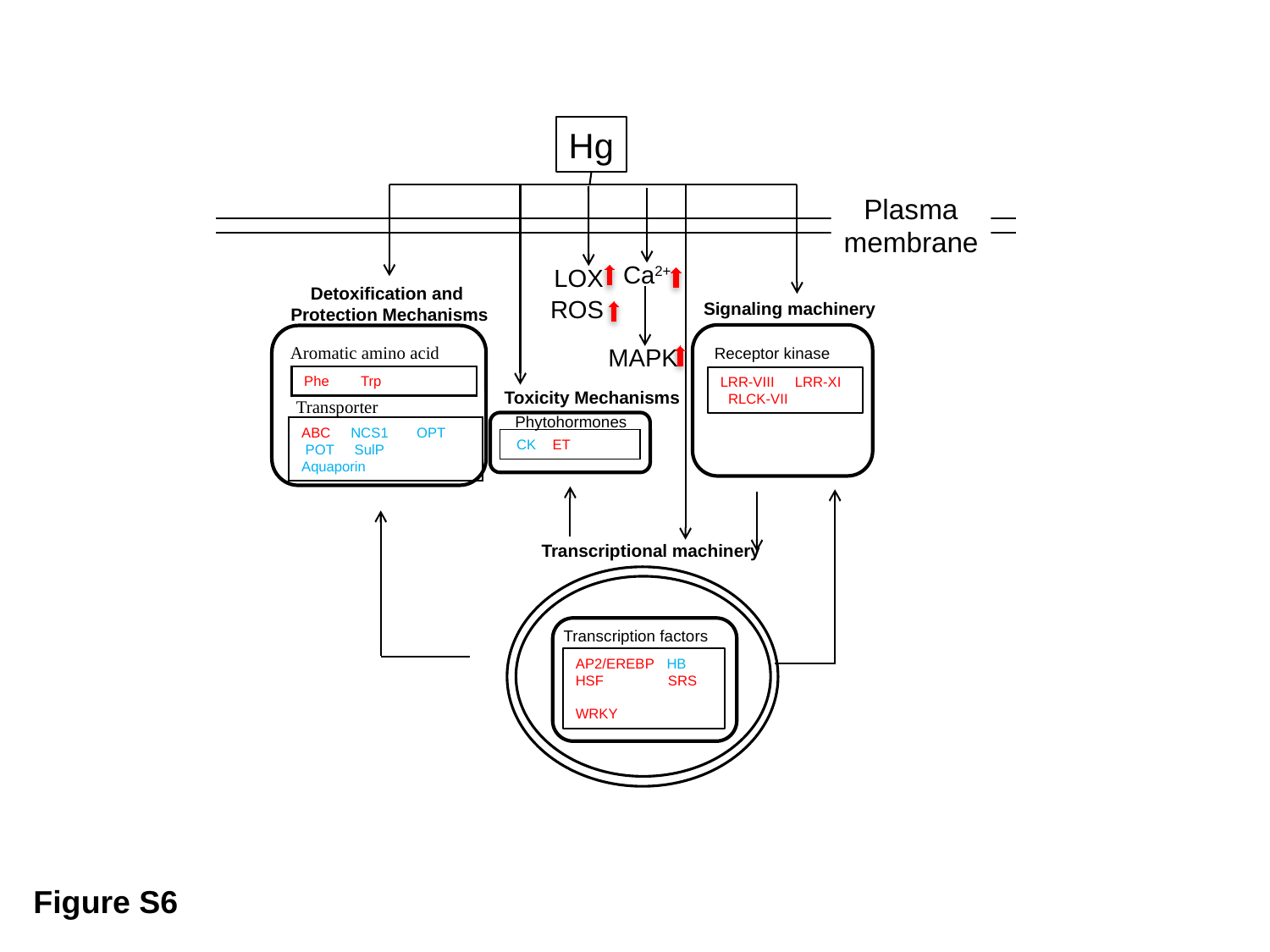

Hg
Plasma
membrane
Ca2+
LOX
Detoxification and
Protection Mechanisms
ROS
Signaling machinery
Aromatic amino acid
MAPK
Receptor kinase
Phe Trp
LRR-VIII LRR-XI RLCK-VII
Toxicity Mechanisms
Transporter
Phytohormones
ABC NCS1 OPT POT SulP Aquaporin
 CK ET
Transcriptional machinery
AP2/EREBP HB HSF SRS
WRKY
Transcription factors
Figure S6
