# Supplementary material for: Transcriptome Profiling and Physiological Studies Reveal a Major Role for Aromatic Amino Acids in Mercury Stress Tolerance in Rice Seedlings
Source: PLoS One. 2014 May 19;9(5):e95163. doi: 10.1371/journal.pone.0095163 (PMC4026224; doi:10.1371/journal.pone.0095163)
Supplement: Table S5 — Hg-responsive transcripts related to transporter genes and phytohormone-related genes. (DOCX) [file pone.0095163.s006.docx]

**Table S5** Hg-responsive transcripts related to transporter genes and phytohormone-related genes

|  | |  | | Short-term Hg exposure,  1 to 3 h | | | Long-term Hg exposure,  24 h | |
| --- | --- | --- | --- | --- | --- | --- | --- | --- |
| Functional categories | | No. of genes in rice genome | | Expression increased | | Expression decreased | Expression increased | Expression decreased |
| **Transporter** | | | | | | | | |
| **ATP-dependent** | | |  | |  |  |  |  |
| ATP-binding cassette superfamily^＊＊^ | | | 130 | | 20 | 0 | 10 | 1 |
| **Secondary transporter** | | |  | |  |  |  |  |
| Proton-dependent oligopeptide transporters^＊＊^ | | | 86 | | 5 | 2 | 2 | 8 |
| Nucleobase:Cation Symporter-1^＊^ | | | 1 | | 0 | 1 | 0 | 0 |
| Sulfate permeases^＊^ | | | 14 | | 3 | 3 | 1 | 0 |
| Oligopeptide Transporter^＊^ | | | 29 | | 0 | 3 | 0 | 2 |
| **Phytohormone** | | | | | | | | |
| Cytokinin^＊^**^＊^** | **Total** | | **54** | | **1** | **1** | **1** | **7** |
|  | Biosynthesis | | 11 | | 1 | 0 | 0 | 0 |
|  | Deactivation | | 11 | | 0 | 0 | 1 | 2 |
|  | Signaling | | 32 | | 0 | 1 | 0 | 5 |
| Ethylene^＊^**^＊^** | **Total** | | **29** | | **5** | **1** | **2** | **1** |
|  | Biosynthesis | | 13 | | 5 | 0 | 2 | 1 |
|  | Signaling | | 16 | | 0 | 1 | 0 | 0 |

Number of genes with significant differences (FDR <0.05) in transcript abundance. Transporter and phytohormone-related gene families that are overrepresented are shown with asterisks (P<0.05).
